# A Case of Enigmatic Stroke Caused by Severe Systemic Sepsis: The Importance of Careful Assessment of Insidious Systemic Infection

**DOI:** 10.7759/cureus.26537

**Published:** 2022-07-03

**Authors:** Taijun Hana, Raj S Lavadi, Ryoko Niwa, Sho Nakamura, Soichi Oya

**Affiliations:** 1 Department of Neurosurgery, Saitama Medical Center/University, Kawagoe, JPN; 2 Department of Neurosurgery, Vydehi Institute of Medical Sciences and Research Centre, Bengaluru, IND

**Keywords:** psoas position, stroke, sepsis, physical examination, iliopsoas abscess, central nervous system infection

## Abstract

Severe sepsis is a dreaded disease with high mortality, especially in the case of delayed detection. Early diagnosis and treatment initiation is critical for patient survival. However, the septic conditions might be masked by other clinical conditions such as stroke, which may result in a serious delay in diagnosis and treatment. We report a case of iliopsoas abscess that initially presented with cerebellar infarction and subarachnoid hemorrhage. Although severe neurological symptoms were prominent, some signs indicating systemic infection, such as “psoas position”, prompted us to investigate the existence of systemic infection. Consequently, severe sepsis with multiple infectious foci, such as iliopsoas abscess, purulent spondylitis, mitral valve valvulitis, and brain abscess, was revealed and was detected as the cause of stroke. The timely and accurate diagnosis of sepsis minimized the delay of the initiation of antibiotic treatment. Approximately five months of intensive care, including two heart valve surgeries, cured the patient, and she was discharged with no neurological deficit. This case demonstrates the importance of careful assessment of the insidious systemic infection as a covert cause of stroke.

## Introduction

Sepsis is a critical condition recognized as a systemic inflammatory response syndrome with an infectious source [1]. Severe sepsis is one of the most challenging diseases to treat even in modern times, as its fatality rate can be over 28% [2]. Early detection and intervention are critical as every one h delay of antibiotic initiation decreases survivability by 7.6% [3]. However, prompt diagnosis is not always possible since sepsis presents with nonspecific and multisystemic involvement. Neurological disorders may be the primary manifestation, making the infection inconspicuous [4]. Here, we present a case of complicated stroke caused by sepsis due to multiple systemic infections.

## Case presentation

**Figure 1:**
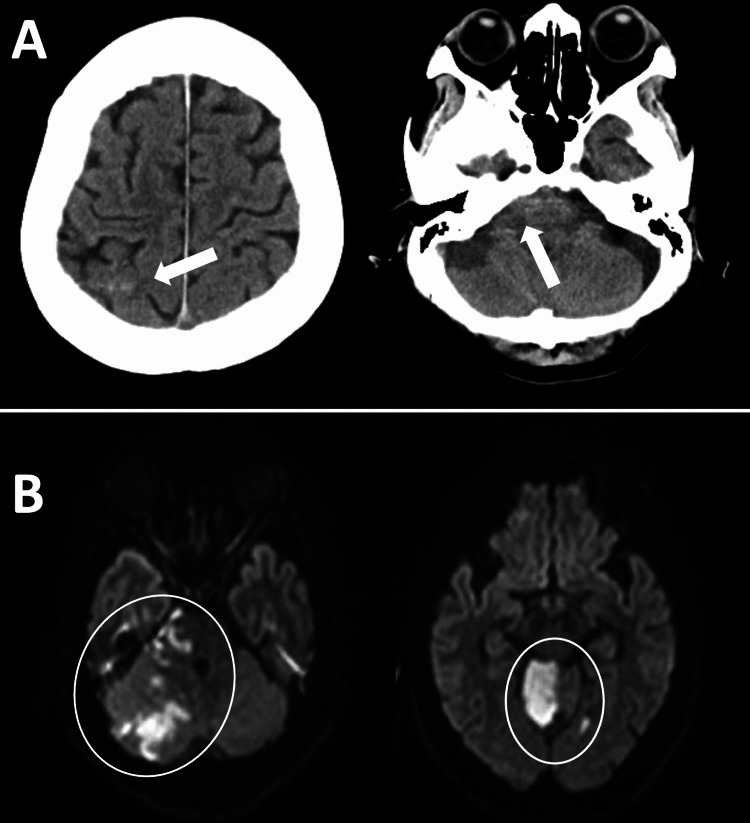
Neurodiagnostic images A) The non-enhanced computed tomography image on admission showed a high-density area (HDA) considered to be subarachnoid hemorrhage (arrows). The HDA of the left image was confirmed as subarachnoid hemorrhage. However, the HDA of the right image was confirmed as an intracranial abscess lately. B) Magnetic resonance imaging diffusion-weighted image of cerebellar infarction on admission (circled).

A 71-year-old woman with a recent history of urinary tract infection (UTI) presented to our neurosurgery service for disturbed consciousness. Other than the UTI, she had no particular medical history, including lifestyle diseases. On admission, she had spastic dysarthria and right-sided cerebellar ataxia as well as mildly disturbing consciousness (E3V4M6 on the Glasgow Coma Scale). Neurological examination showed bilateral (right > left) lower limb weakness, and Babinski’s and Chaddock’s signs were positive on her left side. Although her pupillary reflex was normal, her drowsiness interfered with the detailed cranial nerve examination. The results of the electrocardiogram, blood glucose level, and lipid profiles were normal and showed no risk factors related to stroke. The computed tomography (CT) scan showed a high-density area considered to be a subarachnoid hemorrhage, mainly localized to the right cerebellopontine cistern and in the right parietal convexity (Figure 1A). The magnetic resonance imaging demonstrated multiple acute infarctions in the right cerebellum, cerebellar peduncles, and lateral pons (Figure 1B). The angiography showed that there was an interruption in the middle of the right anterior inferior cerebellar artery and the right superior cerebellar artery. Sinus thrombosis was not observed. Conservative management of stroke was started.

**Figure 2:**
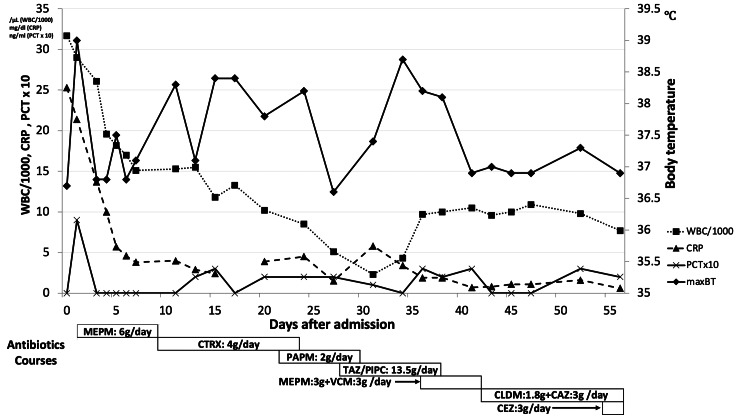
Course of treatment before the cardiac surgery Left vertical axis: WBC/1000 (/μL), CRP (mg/dl), PCT x 10 (ng/ml); right vertical axis: body temperature (℃); horizontal axis: days after admission BT: body temperature, CRP: C-reactive protein, PCT: procalcitonin, WBC: white blood cell, MEPM: meropenem, CTRX: ceftriaxone, PAPM: panipenem, TAZ/PIPC: tazobactam/piperacillin, VCM: vancomycin, CLDM: clindamycin, CAZ: ceftazidime, CEZ: cefazolin.

Although the patient had no fever upon admission, her laboratory tests on admission revealed that her initial white blood cell (WBC) count and C-reactive protein (CRP) level were highly elevated (Figure 2). We conducted extensive bacterial culture screening of the urine, sputum, cerebrospinal fluid, and blood immediately after she was admitted. Although she had a high fever (39 °C) on the next day of admission, it was initially considered to be a stroke-induced inflammatory reaction. A detailed blood examination (WBC: 29000/µL, CRP: 21. 4 mg/dL, platelets: 106000/µL, prothrombin time-international normalized ratio: 1.22, activated partial thromboplastin time: 53.0 s, D-dimer: 7.37 µg/mL, blood urea nitrogen: 32 mg/dL, creatinine: 0.89 mg/dL, procalcitonin: 0.9 ng/mL) suggested the existence of systemic sepsis. 

**Figure 3:**
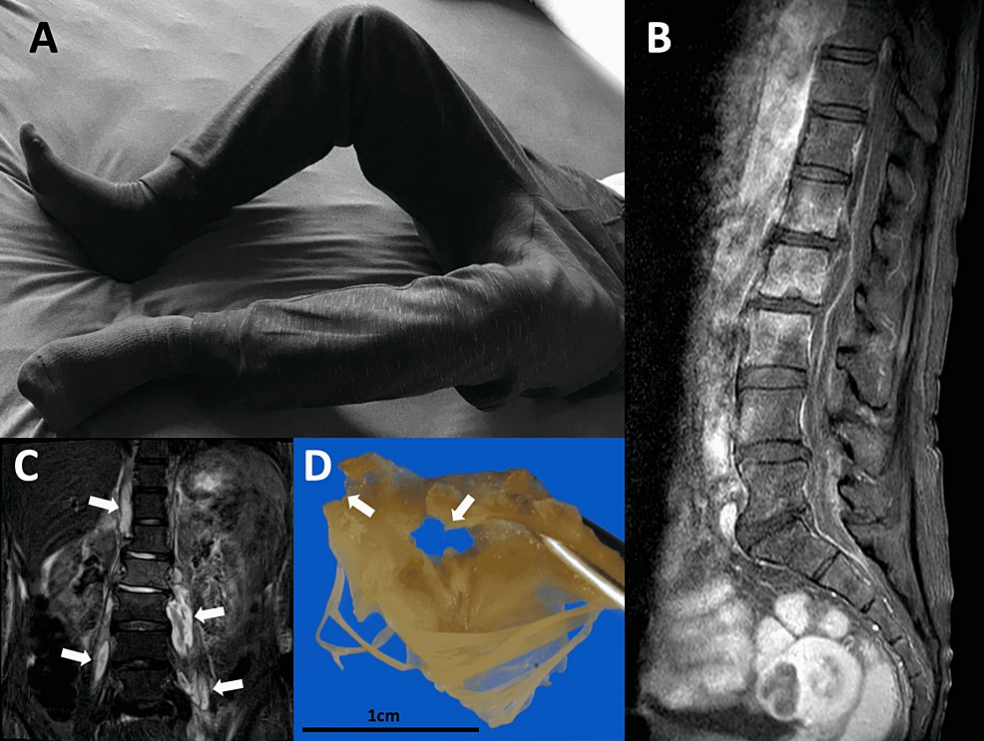
Multiple diagnostic images A) An example of “psoas position”. The patient remains in a hip-joint flexion position. This is a reproduced view of the actual patient’s limb position. B) Magnetic resonance imaging (MRI) enhanced T1 image of the spine. L1-L3 area shows purulent spondylitis. C) MRI enhanced T1 image of the bilateral iliopsoas abscess (arrows). D) The resected mitral valve disrupted by infected valvulitis. Arrows indicate the perforations of the valve.

Besides, a meticulous physical examination for fever revealed that her lower limbs were stiff and in a flexed position, which appeared irrespective of the stroke (Figure 3A). This odd limb position prompted us to conduct imaging studies of lumbar areas, and they revealed bilateral severe iliopsoas abscesses and pyogenic lumbar spondylitis with spinal extradural abscess (Figures 3B, 3C). Empirical treatment with meropenem was initiated based on these findings. On day two, CT-guided iliopsoas abscess drainage was performed. Also, methicillin-susceptible *Staphylococcus aureus* (MSSA) was identified from the urine culture on admission. On day four, MSSA was identified from the iliopsoas abscess drained culture as well. This result suggested that her systemic infection primarily started from UTI and it proceeded through pyelonephritis. The antibiotics were changed according to the results of the antibiotic sensitivity test on day eight (Figure 2, lower part). Echocardiographic screening detected severe mitral regurgitation due to infectious mitral valve disruption. Radiological resolution of the brain, spinal, and iliopsoas abscesses was confirmed after multiple antibiotic courses for six weeks. Her neurological deficits, such as dysarthria and ataxia, gradually improved. Care was then taken by the cardiothoracic surgery team, who performed a two-staged valve replacement. Multiple disruptions caused by infection were confirmed in the mitral valve (Figure 3D). The postoperative course was uneventful. After 19 weeks of intensive management, the patient was discharged to a rehabilitation facility with no neurological deficit. Continuous administration of antibiotics to prevent recurrence of the infection will also be continued appropriately.

## Discussion

**Figure 4:**
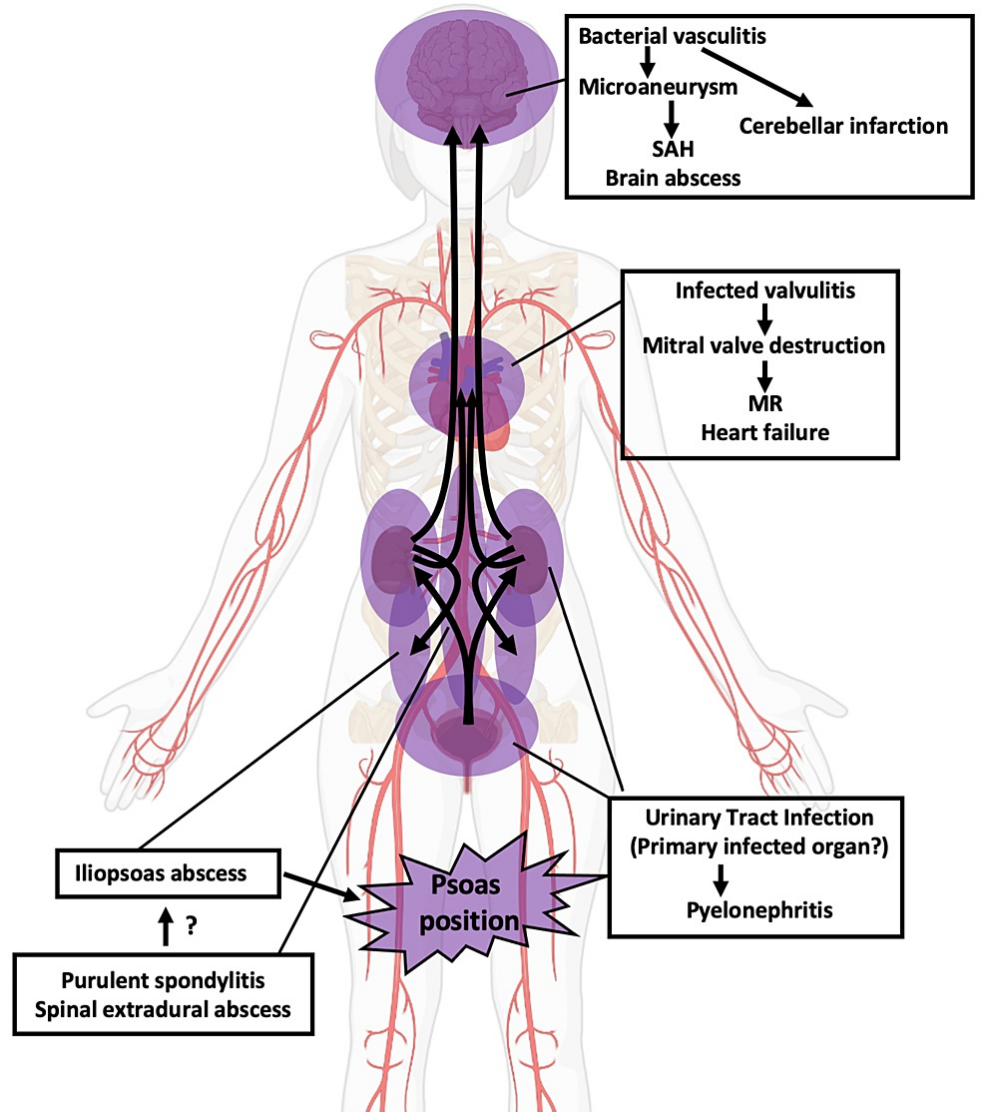
The suggested route of infection SAH: subarachnoid hemorrhage, MR: mitral regurgitation

We reported a case of sepsis with the main clinical manifestation of stroke. The suggested route of infection is illustrated in Figure 4. Atypical symptomatology in elderly patients may lead to a delay in the treatment of underlying sepsis [5]. Early symptoms of a stroke are frequently accompanied by inflammatory reactions due to concomitant infections such as aspiration pneumonia or UTI [6]. Laboratory tests and radiological examinations are important to diagnose infection; however, our case reiterates the importance of careful physical examination in the diagnostic process to promptly determine the source of systemic infection. Meticulous physical examinations during the initial evaluation phase would contribute to timely implementation and accurate interpretation of image screening, such as whole-body CT scanning. In the present case, we noticed the odd posture of the patient’s lower limbs during the neurological examination, which led to a lumbar imaging study. This position has previously been reported as the “psoas position”; the mechanism of this phenomenon is considered to be an unconscious reaction to avoid the pain by stretching the inflamed psoas muscle when the abscess exists around the psoas muscle [7-9].

Urgent antibiotic therapy is mandatory in the treatment of sepsis, for up to 11.6% of patients with sepsis are misdiagnosed in emergency departments. A previous report showed that 63% of medical misdiagnoses were attributable to inadequate physical examinations [10, 11]. According to a large-scale cohort study using the American social medical database, sepsis significantly increases the risk of an ischemic and hemorrhagic stroke, especially within 15 days after the onset of sepsis [12]. Thus, clinicians involved in the care of patients with stroke should explore the cause of possible infection underlying the stroke development.

## Conclusions

In conclusion, it is essential to assess the possibility of coexisting infections in the treatment of patients with stroke. Early therapeutic intervention for infections may improve the outcome of stroke treatment. This case also underlines the importance of careful physical examination in treating patients with stroke.
